# 

**Published:** 1867-02

**Authors:** 


### CONTENTS.

ORIGINAL COMMUNICATIONS.

Histology.................... 45
A comparison of the Present with the Past.................... 57

PROCEEDINGS OF SOCIETIES.

Minutes of the First Annual Meeting of the Ohio State Dental Association.................... 67
Transactions of the Central States Dental Association.................... 76
Dental Meeting.................... 85

EDITORIAL.

Dental Education.................... 86

